# The Role of Salivary and Intestinal Complement System Inhibitors in the Midgut Protection of Triatomines and Mosquitoes

**DOI:** 10.1371/journal.pone.0006047

**Published:** 2009-06-25

**Authors:** Veruska Cavalcanti Barros, Jéssica Góes Assumpção, André Miranda Cadete, Vânia Cristina Santos, Reginaldo Roris Cavalcante, Ricardo Nascimento Araújo, Marcos Horácio Pereira, Nelder Figueiredo Gontijo

**Affiliations:** 1 Department of Parasitology, Federal University of Minas Gerais (UFMG), Belo Horizonte, Minas Gerais, Brazil; 2 Department of Parasitology and Microbiology, Federal University of Piauí, Teresina, Piauí, Brazil; Instituto Oswaldo Cruz and FIOCRUZ, Brazil

## Abstract

Saliva of haematophagous arthropods contain biomolecules involved directly or indirectly with the haematophagy process, and among them are encountered some complement system inhibitors. The most obvious function for these inhibitors would be the protection of the midgut against injury by the complement. To investigate this hypothesis, *Triatoma brasiliensis* nymphs were forced to ingest human serum in conditions in which the protection of midgut by the inhibitors is bypassed. In these conditions, the anterior midgut epithelium was injured by the complement, causing cell death. Once some insects such as *Aedes aegypti* have no salivary inhibitors, we hypothesized the existence of intestinal inhibitors. The inhibitory activity was investigated in the intestine of *A. aegypti* as well as in the saliva and intestine of other three triatomine species (*T. brasiliensis, T. infestans* and *Rhodnius prolixus*) using an immunological method able to determine the level of deposition of some complement factors (C1q, C3b, or C4b) on the surface of complement activator molecules linked to microplates. This methodology permitted to identify which points along the activation phase of the complement cascade were inhibited. As expected, soluble contents of *A. aegypti*'s intestine was capable to inhibit C3b deposition by the classical and alternative pathways. Saliva or soluble intestinal contents, obtained from triatomines were unable to inhibit C1q deposition by the classical pathway. C4b deposition by the classical pathway was inhibited by the intestinal contents from the three triatomines. On the other hand, only *T. brasiliensis* saliva inhibited C4b deposition. Both, saliva and intestinal contents from all triatomines were able to inhibit C3b deposition in the classical and alternative pathways. None of the material extracted from the intestinal cell membranes from the triatomines inhibited C3b deposition in the classical pathway. The existence of complement inhibitors may have important biological consequences which are discussed in detail.

## Introduction

Saliva of haematophagous arthropods posses several molecules involved with the haematophagic process. The main salivary activities are that related with inhibition of vasoconstriction, platelet aggregation and coagulation, i.e., the major physiological processes of host homeostasis [1]. However, the success of the haematophagic process in these organisms also depends indirectly on other activities which necessity are not so obvious. Among these activities, attention should be given to the ones that counteract the host adaptive or innate immune system including specially the complement system [2]–[5].

The complement system is a very important component of the immune defense. It responds promptly to challenges by microorganisms promoting their opsonization by specialized proteins in order to increase phagocytosis and, in a second step, promoting membrane lysis. There are three major routes to complement activation, named Classical, Alternative and Lectin pathways. These pathways converge to a unique sequence of events: the formation of the membrane attack complex (MAC) responsible for membrane lysis [6], [7]. In addition to its role in the innate immunity acting directly on microorganisms, the complement system plays an important role in the normal function of the adaptive immune system, contributing markedly to antigen presentation that makes the humoral response much more efficient [7]–[10].

The ability of saliva on inhibiting the alternative pathway of the complement system was described for the first time in Ixodes ticks [11]. By using hemolytic assays, we have shown that saliva of the triatomines *Triatoma brasiliensis*, *Rhodnius prolixus* and *Panstrongylus megistus* (Hemiptera: Reduviidae) was able to inhibit the classical pathway of the human complement [12]. Conversely, saliva from the mosquito *Aedes aegypti* (Diptera: Culicidae) and from the flea *Ctenocephalides felis* (Siphonaptera: Pulicidae), were unable to inhibit the classical pathway [12]. The inhibition of the alternative pathway by saliva from these haematophagous insects was not investigated. In the same work, we demonstrated the inhibition of the classical pathway by the saliva from the phlebotomines *Lutzomyia longipalpis* and *L. migonei* (Diptera: Psychodidae), and inhibition of the alternative pathway only by *L. longipalpis* saliva.

The presence of anti-complement activity in the saliva of haematophagous arthropods, phylogenetically distinct, suggests that complement-inhibitors may have an important physiological role for these organisms. The most obvious function we could attribute to these inhibitors would be the protection of the cells from the digestive tract against the attack of the complement system after blood ingestion. According to this hypothesis, haematophagous insects such as *Aedes* and *Ctenocephalides* should inhibit the complement system at the digestive tract level to compensate the lack of salivary inhibitors.

All biochemical reactions are specially influenced by the pH of the medium and the complement cascade is not an exception. To our knowledge, there is no precise information in the literature about the operation of the complement system in pHs different from 7.4, which is the normal pH of the extracellular fluids. The blood ingested by mosquitoes and phlebotomine sand flies undergoes alkalization [13], [14] and, significant modifications in the pH of the ingested blood (alkalization or even acidification) would be expected for other haematophagous species. If inhibitors are really necessary for protection of the midgut, the complement should be active in this new environment. Considering these facts, it would be important to know the pH of the blood ingested by an insect under study as well as to investigate the activity of the complement system in different pHs.

In this context, the first objective of the present study was to obtain evidences of the protector role proportioned by the complement inhibitors using as model the triatomine *T. brasiliensis*. The second was to measure the pH inside the anterior midgut of the same insect and to investigate the influence of different pHs in the normal operation of the complement cascade. The third was to search for the existence of anti-complement activity in the digestive tract from the mosquito *Aedes aegypti* as well as from three species of triatomines: *Triatoma infestans, T. brasiliensis,* and *R. prolixus*. Finally, the fourth objective was to characterize the salivary and intestinal anti-complement activities from the three species of triatomines in order to determine which points of the activation step of the complement cascade were affected by the inhibitors.

The existence of complement inhibitors may have important biological consequences regarding a possible interference in the formation of anti-salivary antibodies by the hosts and the survival and development of complement sensitive pathogens in the vectors. These interesting ideas are discussed here in detail.

## Results

### Physiological role of the complement inhibitors in the protection of the digestive tract

The role of complement inhibitors in the protection of the digestive tube was investigated in conditions where the protection performed by the inhibitors was incomplete and, in consequence, the epithelium was attacked. In these assays, the MAC assembling on the membrane of the epithelial cells was evidenced by immunofluorescence.

**Figure 1:**
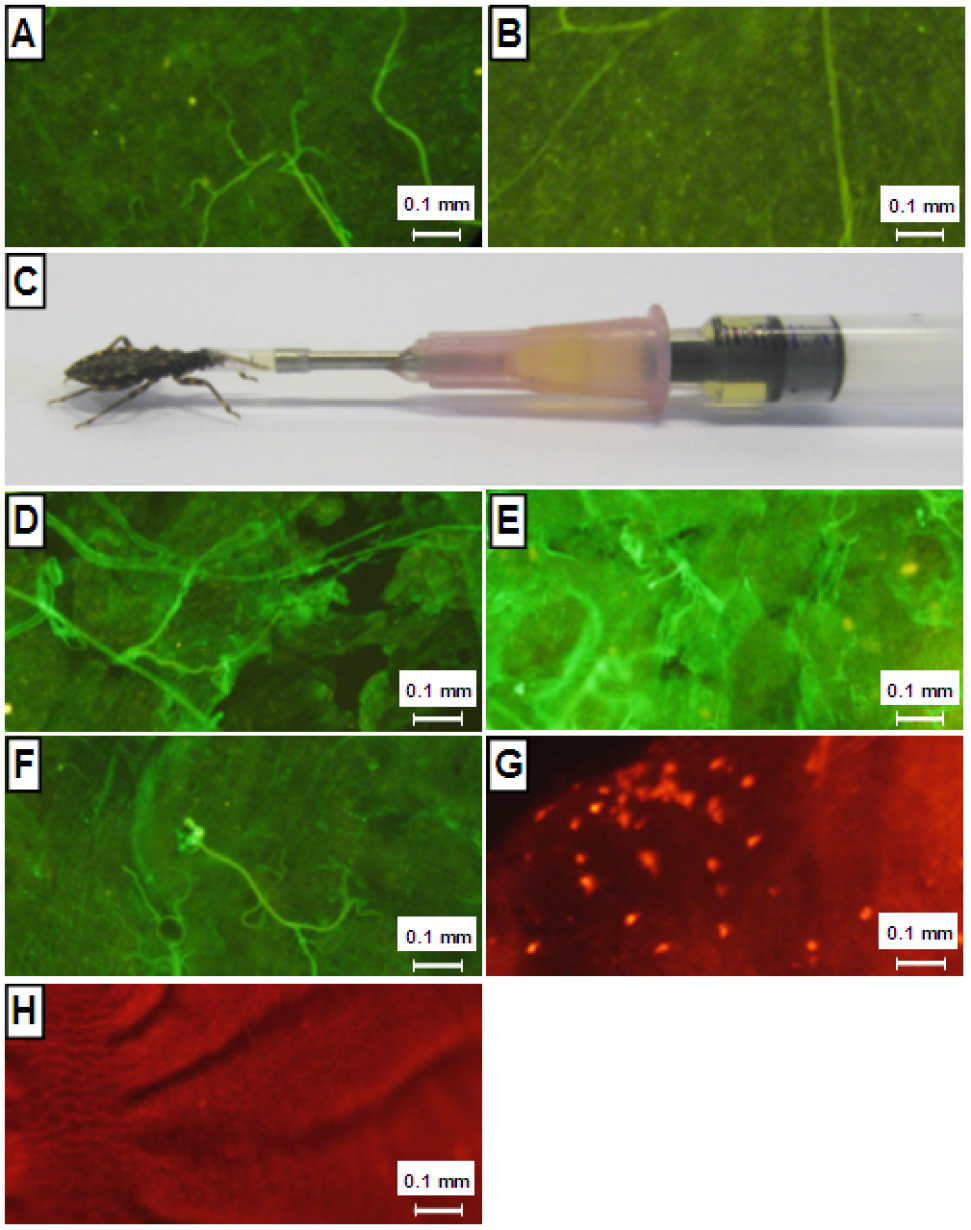
Protection of anterior midgut of *T. brasiliensis* against the complement system. A- MAC deposition onto the anterior midgut wall after normal blood ingestion, B- natural fluorescence observed on the midgut wall, C- Apparatus used for the forced feeding procedure, D- MAC deposition onto the anterior midgut wall after forced feeding of 50 µL of normal human sera, E- Increased deposition of MAC onto the anterior midgut wall after forced feeding of 50 µL of 2 fold concentrated normal human sera, F- MAC deposition onto the anterior midgut wall after forced feeding of 50 µL of inactivated 2 fold concentrated normal human sera, G- Cell death in the anterior midgut epithelium after forced feeding of 2 fold concentrated normal human serum containing propidium iodide. H- Absence of cell death after forced feeding of inactivated 2 fold concentrated normal human sera containing propidium iodide.

**Figure 2:**
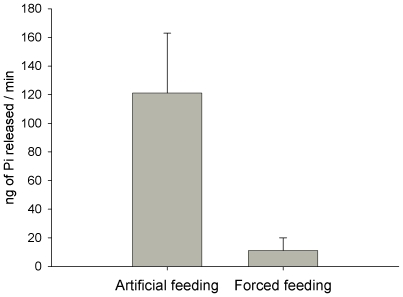
Apyrase activity from the crop soluble contents of *Rhodnius prolixus* after artificial and forced feeding. The activity was expressed as ng of inorganic phosphate (Pi) released in a minute by 1/12 of anterior midgut contents±standard error. The T test indicated a significant difference between groups (P<0.05).

When fourth instar nymphs of *T. brasiliensis* ingested blood from human voluntaries, no marked areas in the anterior midgut (crop) were seen (**Figure 1-A**) except for the natural intestine fluorescence (**Figure 1-B**). In this case, we can attribute the absence of attack to an effective concentration of salivary and intestinal inhibitors acting together inside the anterior midgut (it is important to consider that, in triatomines, saliva is regularly ingested during a blood meal [15]). To investigate the importance of the complement inhibitors in protecting the anterior midgut, we forced the insects to ingest sera in a condition in which saliva ingestion is drastically reduced. This forced feeding procedure was accomplished by using the apparatus shown in **Figure 1-C**. In this condition, practically only intestinal inhibitors would be available to protect the intestinal epithelium and they were not enough to do it. The nearly absence of saliva in the forced fed nymphs was confirmed by using the salivary enzyme apyrase as a saliva tracker (**Figure 2**). According to **Figure 1-D**, the epithelium of the anterior midgut was slightly marked by MAC proteins after the forced feeding with 50 µL of normal human serum. The marking intensity increased considerably when nymphs were forced to feed the same volume of two fold concentrated sera (**Figure 1-E**). No marks were observed when insects were forced to ingest 50 µL of inactivated two fold concentrated human sera (**Figure 1-F**) except for the natural fluorescence similar to that in **Figure 1-B**.

The fluorescent dye propidium iodide was used to access for the death of epithelial cells when exposed to the complement present in the 2 fold concentrated human sera. This dye penetrates dead cells and makes their nucleus fluorescent. In fact, the MAC deposition on the epithelium caused cell death after 1 hr of exposition. **Figure 1-G** shows groups of dead cells marked in red by the propidium iodide dye and, **Figure 1-H** shows the absence of marked areas in insects that ingested inactivated 2 fold concentrated human sera.

### Anterior midgut pH and complement system operation in different pH

The pH measured in the *T. brasiliensis* anterior midgut lumen was 7.16±0.26 (n = 6) during the first two hours after blood ingestion and practically continued the same after 24 hours (7.02±0.05, n = 3). For this study, we assumed that the anterior midgut pH is similar in all triatomine species.

**Figure 3:**
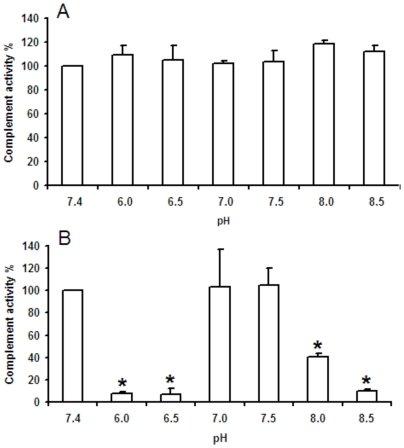
Influence of pH in the operation of the human complement system. Operation of the classical (A) and alternative (B) pathways at different pHs when compared with the control at pH 7.4 (100%).

In order to verify if the complement system can be activated in the anterior midgut of blood fed triatomines and other insects such as mosquitoes and phebotomines, the performance of the human complement was investigated by measuring the level of C3b deposition in the classical and alternative pathways at pHs 6.0 to 8.5 with 0.5 pH unit intervals. These assays were prepared in ELISA microplates as described in the material and methods section. According to **Figure 3-A**, the effectiveness of C3b deposition by the classical pathway was the same in all pHs tested, with no difference when compared to the results obtained at standard conditions i.e. at pH 7.4 (n = 3). On the other hand, the deposition of C3b by the alternative pathway (**Figure 3-B**) was similar to that obtained at standard conditions, only at the pHs 7.0 and 7.5 (n = 3). The effectiveness of C3b deposition, by the alternative pathway, at pH 8.0 was 41±3% (n = 3) of that usually obtained at pH 7.4. The classical and alternative pathways operated equally well between pHs 7.0 and 7.5 and the pH of the ingested blood is between this range, therefore, all inhibition assays were performed in standard conditions at pH 7.4.

### Inhibitory effects over the complement system cascade

The human complement system is triggered when in contact to antibodies (classical pathway) [16] or agarose (alternative pathway) adhered to a microtiter plate [11]. The factors of the complement system, which are activated in cascade and normally adhere to the surface of the plates, can be detected by using antibodies. We modified these assays in order to allow the measurement of the effect of different samples obtained from haematophagous insects over the deposition of the components C1q, C4b and C3b in the classical pathway or C3b in the alternative pathway.

According the results obtained, all the three triatomine species studied were able to inhibit both the classical and the alternative pathways from the complement system. The data generated using saliva and soluble intestinal contents as possible inhibitors are summarized in **Tables 1** **and** **2**, where the inhibitory effects in different points of the complement cascade could be observed. None of the samples obtained from triatomines was able to significantly inhibit C1q from binding the IgG molecules used to trigger the classical pathway, indicating that the inhibition occurs downstream along the cascade. C4b deposition by the classical pathway was inhibited by the soluble intestinal contents from the three triatomine species, however only *T. brasiliensis* saliva was able to inhibit C4b deposition at this point. Both saliva and intestinal soluble contents from the three triatomine species were able to inhibit C3b deposition in the classical pathway. None of the proteins extracted from the anterior midgut membranes from the three triatomine species was able to inhibit the C3b deposition through the classical pathway (p>0.05, n = 3).

**Table 1:** Percentage of inhibition of the complement system by saliva from *Triatoma brasiliensis, T. infestans* and *Rhodnius prolixus*.

Species	Number of salivary lobes per assay	Inhibition of C1q deposition by CP (n)	Inhibition of C4b deposition by CP (n)	Inhibition of C3b deposition by CP (n)	Inhibition of C3b deposition by AP (n)
*T. brasiliensis*	2	5±24 (3)	*46±6 (5)	*34±15 (4)	*92±3 (3)
	1	2±16 (3)	16±9 (5)	22±16 (4)	*89±7 (3)
	0.5	6±2 (3)	6±16 (4)	13±13 (4)	*84±7 (3)
*T. infestans*	2	0±25 (3)	11±21 (7)	*56±5 (3)	*88±9 (3)
	1	11±3 (3)	13±16 (7)	*43±12 (3)	*80±15 (3)
	0.5	15±11 (3)	9±9 (7)	32±13 (3)	*74±18 (3)
*R. prolixus*	2	0±4 (5)	3±7 (3)	*33±23 (3)	*59±23 (5)
	1	0±1 (5)	2±6 (7)	28±25 (3)	*62±24 (5)
	0.5	0±2 (5)	0±8 (7)	25±31 (3)	*57±34 (5)

(n) = number of independent experiments performed for each treatment.

Asterisk (*) indicates statistical difference from control (P<0.05).

AP: Alternative Pathway, CP: Classical Pathway.

**Table 2:** Percentage of inhibition of complement system by the intestinal contents from *Triatoma brasiliensis, T. infestans* and *Rhodnius prolixus*.

Species	Number of salivary lobes per assay	Inhibition of C1q deposition by CP (n)	Inhibition of C4b deposition by CP (n)	Inhibition of C3b deposition by CP (n)	Inhibition of C3b deposition by AP (n)
*T. brasiliensis*	1	17±18 (3)	*51±9 (5)	*77±9 (4)	*80±4 (3)
	0.5	3±16 (3)	27±23 (5)	*60±18 (4)	*71±5 (3)
	0.25	8±14 (3)	13±37 (5)	*49±17 (4)	*46±8 (3)
*T. infestans*	1	22±31 (3)	*57±24 (4)	*51±35 (4)	*66±24 (3)
	0.5	20±34 (3)	29±18 (4)	*57±16 (4)	*47±37 (3)
	0.25	2±1 (3)	19±16 (4)	*45±28 (4)	*53±21 (3)
*R. prolixus*	1	3±3 (4)	*59±18 (3)	*35±14 (3)	*61±4 (3)
	0.5	0±1 (4)	47±29 (3)	33±24 (3)	*40±7 (3)
	0.25	0±2 (4)	34±35 (3)	20±20 (3)	*38±1 (3)

(n) = number of independent experiments performed for each treatment.

Asterisk (*) indicates statistical difference from control (P<0.05).

AP: Alternative Pathway, CP: Classical Pathway.

In the complement cascade, the first step to trigger the alternative pathway consists in the activation of the component C3 by proteolytic cleavage, followed by the C3b deposition onto the activation substrate. This step was promptly inhibited by saliva and soluble intestinal contents from the three triatomine species studied (**Tables 1** **and** **2**). The action of the proteins extracted from the anterior midgut cell membranes over the alternative pathway could not be studied because the technique used suffers interference of the detergent used to solubilize these proteins.

These results indicate that the inhibitors were able to interrupt the complement cascade in the first events of the activation phase, where the inhibition is more effective because, in this case, the amplification of the process is blocked.

The soluble intestinal contents from *T. brasiliensis* and *T. infestans* was ultrafiltered in a 5 kDa cut-off membrane and the resulting material, containing solely low molecular weigh molecules, was able to significantly inhibit C3b deposition by the classical (*T. brasiliensis*: 24±12%, *T. infestans*: 18±2%; p<0.05, n = 3) and alternative pathways (*T. brasiliensis*: 30±6%, *T. infestans*: 66±12%; p<0.05, n = 3). The quantity of ultrafiltered material used in the assays was approximately equivalent to intestinal contents of two insects and the total protein concentration was 11±6 µg/midgut for *T. brasiliensis* and 23±8 µg/midgut for *T. infestans*.

The possible existence of a complement system inhibitor in the haemolymph from the three triatomine species studied was also investigated. None of them inhibited the C3b deposition by the classical or alternative pathways. In addition to triatomines, the digestive tract from the mosquito *A. aegypti* was tested for inhibitory activity. A preparation containing midgut soluble molecules from two females was able to significantly inhibit C3b deposition by the classical (24±5%, n = 3, p<0.05) and alternative (52±11%, n = 3, p<0.05) pathways.

## Discussion

In hemipterans, the microvilly of the midgut are covered by an unusual structure called perimicrovillar membrane, which extends toward the luminal compartment with a dead end [17]. The perimicrovillar membrane is well developed in fed insects but is poorly developed in unfed ones [17]. Although the perimicrovillar membrane may confer some level of protection to the triatomine digestive tube, this protection was not sufficient to prevent the complement attack against the epithelium in order to avoid cell death (**Figure 1**).

Differently from the triatomines, the mosquito *A. aegypti* secrets a peritrophic matrix (PM) around the blood bolus localised in its posterior midgut. Someone could speculate that PM could be protective against the complement but, according Devemport and Jacobs-Lorena [18] the PM in *A. aegypti* is first detected 4–8 hr after blood ingestion and becomes mature only 12 hr post-feeding. In consequence, its gut epithelium is extensively exposed to complement factors just after the blood intake and requires the action of inhibitors.

It is well known that during blood ingestion in live hosts, saliva is ingested combined to the blood [19]. The ingestion of saliva was specially demonstrated in *Rhodnius prolixus* [15] by measuring the apyrase activity in the crop after feeding. Apyrase is an enzyme only found in the saliva and the activity in the crop can be used to estimate the amount of saliva ingested during the feeding. When the insects were allowed to feed on humans under normal conditions, the combined action from salivary and intestinal inhibitors was sufficient to prevent the attack to the epithelium (**Figure 1-A**). The forced feeding procedure, used to obligate *T. brasiliensis* fourth instar nymphs to ingest human active sera, was carried out in such a way that saliva ingestion would be drastically reduced as occurs with *R. prolixus* (**Figure 2**). Under this specific circumstance, the intestinal inhibitors were acting almost alone in the intestinal environment. When the insects were forced to ingest two fold concentrated human sera, the inhibitors inside the midgut were not sufficient to prevent the intestinal epithelium from the complement attack. The epithelium was then strongly marked with anti-C5-C9 antibodies (anti-MAC) (**Figure 1-E**) confirming that in this circumstance, the complement system is triggered and MAC is formed onto the epithelium. As expected, the ingestion of concentrated sera provoked cell death. Such fact was evident with the appearance of regions marked with propidium iodide (**Figure 1-G**), which has the propriety to penetrate in dead cells and mark their nucleus on red.

The importance of complement system inhibition for haematophagous arthropods was corroborated by the presence of inhibitors in the midgut of the mosquito *A. aegypti*. According to our hypothesis, the haematophagous arthropods that have no salivary inhibitors should have an inhibitory activity at the digestive tract level, as seen for *A. aegypti*. Indeed, the intestinal contents from this mosquito were able to inhibit C3b deposition for both classical and alternative pathways.

It is interesting that the existence of anti-complement activity in *Ixodes scapularis* gut was inferred because the host complement did not suppress the growth of the complement sensitive spirochete *Borrelia burgdorferi* in the digestive tract of ticks after blood feeding [20]. As seen for ticks, we are hypothesizing here that the presence of inhibitory molecules in the saliva and intestine from triatomines could benefit the development of the parasite *T. cruzi*. Trypomastigote forms of this parasite, found in the bloodstream, are resistant to the complement. After a blood meal these forms differentiate, in the anterior midgut of the vector, to epimastigote forms which are very sensible to the complement attack [21]. Considering that differentiation to epimastigotes starts a few hours after ingestion of the parasites [22], it is reasonable to hypothesize that they depend on the complement inhibitors to survive in the vector.

Assuming that complement inhibitors may protect some pathogens, it is reasonable to infer that host antibodies, directed against complement inhibitors (inactivating them), could impair the development of these pathogens. Probably, it could be possible in the future to use complement inhibitors as part of vaccines designed to block the development of complement sensitive pathogens in their vectors.

The complement system normally operates at pH 7.4 that is the normal pH from blood and extracellular fluids. However, in *T. brasiliensis*, and probably in other triatomines, the ingested blood is slightly acidified from pH 7.4 to 7.16 in the anterior midgut lumen, which is the storage organ in these insects. To our knowledge, there is no information about the complement system operation at pHs different from 7.4. If the complement is really dangerous to the insects in order to “force” them to produce inhibitors, complement should be active in the midgut conditions such as in pHs around 7.0 in triatomines and even in alkaline environments as in the abdominal midgut from some mosquito species (including *A. aegypti*) where the pH reaches values equal or higher than 8.0 after a blood meal [13]. Once the intestinal epithelium from insects has a unique cell layer [23], the damage caused by complement activation could lead to the rupture of the digestive tract and even death of the insect. The results obtained here about the performance of the complement system at different pHs show that the classical and alternative pathways are active at the pH 7.16, inside the anterior midgut from triatomines, and even at pH∼8.0, inside the midgut of mosquitoes (**Figure 3**). It is possible that, under these circumstances, the alternative pathway would be triggered by carbohydrates from the glycocalix of the intestinal cells and that the classical pathway would be triggered by unspecific binding of natural antibodies to these carbohydrates or other intestinal molecules. The presence of carbohydrates covering the intestinal membranes could trigger the lectin pathway by binding the MBL protein.

**Figure 4:**
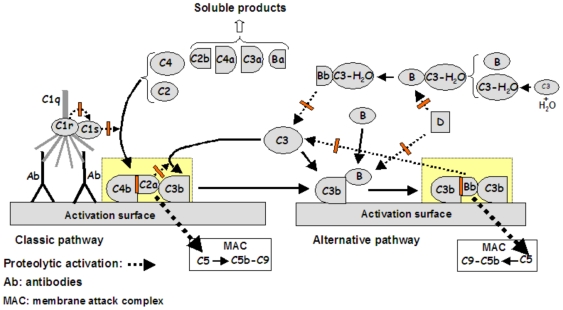
Scheme of the activation phase of the complement system showing the points potentially targeted by the inhibitors. The classic pathway is initiated by the binding of C1q, C1r and C1s (the C1 complex) to antibodies linked to the activation surface. The proteolytic activity of C1r is automatically activated by interaction with C1q. C1r then cleaves and activates C1s, which is another serine protease. C1s acts specifically on C2 and C4 activating them. Once cleaved, the fragment C4b is capable to bind covalently to the activation surface creating a binding site to C2a. The active serine protease C2a, in the C4b-C2a complex, acts on C3 producing C3b molecules which are also capable to bind covalently to the activation surface nearby its site of activation. The complex C4b-C2a-C3b acts as a C3 convertase as well as a C5 convertase generating the MAC. For activation of the alternative pathway, a small fraction of C3 present in the extracelular fluids slowly undergoes spontaneous reaction with H_2_O molecules generating C3-H_2_O. These molecules can interact with the protein B generating the B-C3-H_2_O complex which is a substrate for D, a plasmatic serine protease. The Bb-C3-H_2_O complex acts on C3 cleaving it to C3b and C3a. Most of the C3b molecules generated will combine with H_2_O or other self molecules becoming inactive. On the other hand, if a C3b molecule is generated near an adequate surface such as a bacterium, it will covalently bind to it creating a binding site for factor B. The C3b-B complex is activated by the protease D generating C3b-Bb, an efficient protease capable to activate other C3 molecules. The complex C3b-Bb-C3b is an efficient C3 convertase as well as a C5 convertase generating the MAC. To simplify, the lectin pathway as well as the normal regulatory proteins were omitted.

**Figure 4** contains a scheme of the complement activation process in both, classical and alternative pathways and shows the MAC formation by the action of C3 convertases. C3 convertases also operates as C5 convertases. To the sake of simplicity, the activation of the lectin pathway was omitted. The Mannam-Binding Lectin pathway (also called the MBL pathway) is not activated by any of the protocols used for the inhibitory assays in the present work. Therefore, the results obtained had no influence from this pathway. The red marks in the scheme indicate the most probable points where the salivary and/or intestinal inhibitors may be acting along the complement cascade taking into account the results observed in **Tables 1** **and** **2**.

The inhibition of the C4b deposition in the classical pathway indicates a possible action over C1r and/or C1s, by inhibiting their proteolytic action (C1r activating C1s, or C1s activating C4) as is performed by the soluble C1 inhibitor present in the normal sera [24]. The blockage of C3b deposition in the classical pathway could be attributed to the action of the inhibitors in any point mentioned before or in the C2a proteolytic activity over C3. The presence of any factor accelerating the C2a decay from the C4b-C2a-C3b complex could also lead to the same result. An inhibitor able to accelerate the decay of C2a would be similar to the C4 binding protein (C4bp), which is found soluble in the normal sera. C4bp accelerates the C2a decay from the C4b-C2a-C3b complex and acts as cofactor in the cleavage of C4b by factor I, another soluble regulatory protein [25].

The inhibition of C3b deposition in the alternative pathway could be explained by the direct inhibition of protease D. Protease D is a soluble enzyme, already active in the blood, which has higher specificity to activate factor B, by proteolysis, when it is associated to C3b opsonized to the activator surface or when it is part of the soluble complex B-C3-H_2_O. The activated factor B (Bb) is another protease able to activate C3 to C3b and C3a. Bb inhibition could also promote reduction in C3b deposition onto activator surfaces. Any molecule, present in saliva or intestinal contents from insects, acting over the C3b-Bb-C3b complex and accelerating the decay of Bb would also favour the reduction of C3b deposition in the alternative pathway.

Although the material obtained from midgut microvillosities are not able to inhibit C3b deposition by the classical pathway, we can not discard the possible presence of a MAC-assembling inhibitor inserted on the midgut membranes, as observed on erythrocytes or on the surface of cells naturally exposed to the complement proteins [26].

In addition to the digestive tract protection performed by the salivary and intestinal inhibitors, the complement inhibitory activity found in the saliva could be also directly involved in the modulation of the immune system from vertebrate hosts. Indeed, it's known that even soluble antigens, when opsonized by C3b, are 10 to 100 fold more efficient at inducing humoral responses than when not opsonized [27]. In fact, Dempsey et al. [28] stated that the efficiency of C3b opsonized antigens could increase up to 10,000 fold if the target antigen is doubled marked by C3b. The antigen opsonization (even soluble antigens) with C3b acts as an “adjuvant” and makes the molecules much more efficiently presented to the immune system generating a long lasting secondary response and antibody production with high specificity and affinity [8], [29], [31].

The presence of salivary inhibitors able to act in the activation phase of the complement system would considerably reduce the amount of salivary antigens opsonized with C3b and hence affect the presentation of these antigens to the host immune system. In consequence, the hosts would be less efficient to perform a humoral immune response against salivary proteins preserving their activities which are necessary for a normal haematophagy. In addition to the complement inhibitory activity, the saliva from haematophagous insects has several other activities that affect the immune system [2]–[5], [32]. All of them are in accordance to the idea presented above about the role of immune system inhibitors in the preservation of essential salivary activities.

With time, these mechanisms would be over passed and an immune response starts to appear, at least against the most immunogenic salivary proteins. Despite the final development of a humoral response, this response is in fact not so intense. Although *L. longipalpis* saliva comprises at least 35 different proteins [33] the number of proteins recognized on immunoblots performed with sera obtained from people or animals naturally or experimentally exposed to the insects is considerably low [34]–[39].

## Materials and Methods

### Ethics Statement

This study was conducted according to the Ethical Principles in Animal Experimentation. The study was approved by the Ethics Committee in Animal Experimentation (CETEA/UFMG) under the protocol number 116/2008.

### Insects

Fourth instar specimens of *Triatoma brasiliensis* (Brazil), *T. infestans* (Bolivia) and *Rhodnius prolixus* (Honduras) were obtained from colonies maintained at 28°C and 65% relative humidity. They were fed weekly on chickens (*Gallus gallus*) and rats (*Rattus norvegicus*). The mosquito *Aedes aegypti* was maintained according to the rearing protocol of Eiras and Jepson [40] in a closed colony at 27°C and 70% relative humidity.

### Serum

Pools from normal human sera were obtained from at least 6 health voluntary donors. The material was aliquoted and maintained at −80°C until use.

### Salivary gland extracts

Triatomine saliva was prepared from 12 salivary globules that were dissected, washed in saline (0.9% NaCl) and added to 480 µL of HMEBN (5 mM HEPES/NaOH, 7 mM MgCl_2_, 10 mM EGTA, 140 mM NaCl, 5 mg/mL BSA, pH 7.4) for the alternative pathway assay, or in equal volume of HNCM (4 mM HEPES/NaOH, 145 mM NaCl, 2 mM CaCl_2_, 1 mM MgCl_2_, pH 7.4) for the classical pathway assay. The *R. prolixus* salivary gland has two main globules that were used as saliva source. The *Triatoma* salivary gland is more complex. The subunits D1 and D2 were considered as a single globule and each insect has two of these globules [41]. The preparations were sonicated for 5 sec and centrifuged at 10,000 g for 3 min at room temperature. *A. aegypti* saliva was not investigated.

### Soluble intestinal contents

Each soluble intestinal contents sample was prepared from a pool of 20 midguts from blood unfed *A. aegypti* females with at least 7 days old, or a pool of 6 anterior midguts from each triatomine species obtained from starving fourth instar nymphs with 10 to 20 days after moult. After they were washed in saline, the *Aedes* midguts were transferred to 400 µL of HMEBN or HNCM (to assay the alternative or classical pathway, respectively) and the intestines of triatomines to 480 µL of the same solutions. The *Aedes* material was sonicated for 10 seconds and the triatomine anterior midguts were opened with stylets in order to collect their contents. After centrifugation at 10,000 g for 3 min at room temperature, adequate amounts of the supernatant from each sample were used in the assays. In some experiments, the intestinal contents from *T. infestans* and *T. brasiliensis* were ultrafiltered with a 5 kDa cut-off and the inhibitory activity of the filtered material was assayed.

### Purification of the intestinal microvillosities

Preparations containing a mixture of intestinal microvillosities and perimicrovillar membranes were obtained from the triatomine anterior midgut according to Abdul-Hauf et al. [42] modified. Six anterior midguts from each triatomine species were dissected, opened and washed in saline to completely remove the intestinal contents. The anterior midguts were then transferred to 100 µL of ice-cold MET solution (300 mM mannitol, 5 mM EGTA, 17 mM TRIS-base/HCl, pH 7.5) in a microcentrifuge tube and manually homogenized with an abrasive glass microhomogenizer during 5 min. 100 µL of ice-cold 24 mM MgCl_2_ were added to this preparation and the tube content was mixed. After 20 min on ice, the material was centrifuged at 2,500 g for 15 min at 4°C. The supernatant was collected in another tube and the pellet was homogenized once more in newly added 100 µL of ice-cold MET solution and centrifuged. After repeating this procedure three times, the supernatants were mixed and centrifuged at 25,000 g for 30 min at 4°C. The final pellet, enriched with microvillosities, was dissolved in HNCM containing 0.025% sodium deoxycholate and used for the classical pathway assays.

### Haemolymph

Haemolymph from the 3 triatomine species was collected from fourth instars using the following procedure: nymphs had their femur cut and the leaking haemolymph was collected by using a pipette, transferred to a microcentrifuge tube and centrifuged at 10,000 g for 3 min at room temperature. Twelve microliters of the supernatant were mixed to HMEBM or HCMN to a final volume of 480 µL. Adequate quantities of the preparations were used for inhibitory assays.

### Study of the physiological role of the complement inhibitors in the digestive tract protection

This study was performed by forcing *T. brasiliensis* fourth instar nymphs to ingest human sera with the feeding apparatus shown in **Figure 1-C**. The syringe used in the apparatus was an insulin syringe which permitted a precise control of the volume of material ingested by the nymphs (49±5 µL, n = 13). A plastic tube with approximately 0.5 cm length and 0.58 mm internal diameter was inserted in the head of nymphs (with the proboscides straighten and the antennas cut) and fixed to the insect with a cyanoacrylate-based glue (Super Bonder®). Before connecting the needle to the syringe, the apparatus was carefully filled with the material to be injected avoiding bubbles inside the tube (**Figure 1-C**). With this apparatus, 50 µL of normal sera or 2 fold concentrated sera (active or inactivated by 30 min at 56°C) were injected into the digestive tract of the bug. The 2 fold concentrated sera was obtained in an evaporator centrifuge (CentriVap concentrator-Labconco) at room temperature. After the injection, the insect was allowed to rest for 1 hr and then dissected for collection of the anterior midgut. The anterior midgut was gently opened with an stylet, washed 5 times in PBS and incubated for 20 min with rabbit anti-human C5b-C9 antibody (Calbiochem: 204903) (8,3 µg/µl), diluted at 1∶500 in HNB solution (10 mM HEPES/NaOH, 140 mM NaCl, 1 mg/mL BSA, pH 7.4). After the incubation, the anterior midgut was washed 5 times in PBS and incubated with goat anti-rabbit IgG conjugated with fluorescein (Calbiochem: 401319) 1∶1000 in HNB for 20 min. A new round of washing was performed and the deposition of MAC, now marked with fluorescein, was examined in an Olympus epifluorescence microscope model BX41TF (450–490 nm excitation/535 nm emission). As control, the intestines of some insects that ingested blood naturally from voluntary humans were submitted to the same treatment. The midguts were maintained open by covering them with cover slips.

The fluorescent dye propidium iodide was used to access the death of epithelial cells exposed to the complement from the 2 fold concentrated human sera. Starving fourth instar nymphs were forced to ingest 50 µL of 2 fold concentrated human sera containing 2.5 µg/ml of propidium iodide. After 1 hr resting, the digestive tract was dissected and taken entirely to the epifluorescence microscope (510–560 nm excitation/590 nm emission). The fluorescence was compared to insects that ingested inactivated 2 fold concentrated human sera. Each experiment was performed 3–5 times.

### Evaluation of the saliva ingestion during the forced feeding procedure

The apyrase activity in the crop of *R. prolixus* was used as an indicative of the saliva ingestion in the anterior midgut after the forced feeding procedure. Three groups were tested: after the forced feeding, after natural feeding and unfed fourth instar nymphs. After the treatments, soluble intestinal contents from the anterior midgut were extracted in saline solution (0.9% NaCl) and transferred to ice-cold microcentrifuge tubes containing 30 µl of 20 mM HEPES/100 mM NaCl buffer pH 7.4. Tubes were centrifuged for 3 min at 12,000 g and the supernatant was transferred to a new tube. For the assay, 1/12 of the samples were added to HEPES/NaCl buffer pH 7.4 to a final volume of 20 µl. These 20 µl aliquots were added to 80 µl HEPES/NaCl buffer pH 7.4 containing 4 mM ATP and 5 mM CaCl_2_ and incubated at 37°C for 25 min [15]. After incubations, inorganic phosphate was immediately measured using a commercial kit (Labtest Diagnóstica, Brazil) [43]. The phosphate encountered in unfed nymphs was subtracted from that measured in the natural or forced fed nymphs and the apyrase activity was expressed as nanograms of phosphate released from ATP during a minute.

### Complement system inhibition assays

#### Alternative pathway assay [modified from ref. [11]]

The wells of a microtiter plate (COSTAR® code 9017) were covered with 0.1% agarose (Promega) dissolved with distilled water (100 µL/well). The agarose solution was dried by incubating the plate for 12 hr at 37°C. Ten microliters of normal human sera was pre-mixed inside microcentrifuge tubes, immediately before the assays, to adequate amounts of the samples containing the inhibitors (salivary gland extracts, intestinal contents or haemolymph). The final volume was adjusted to 100 µL with HMEBN. Controls were prepared in separate tubes by adding sera and the HMEBN solution alone. Each one of these treatments was carried out in triplicate. One hundred microliters of each preparation was then transferred to microplate wells containing agarose and incubated for 30 min at 37°C under agitation (approximately 80 rpm). Under these conditions, the alternative pathway is triggered and the C3b component binds covalently to the agarose surface in a higher or lower level, depending on the absence or presence of any inhibitor. After incubation, plates were washed. The washes were performed 3 times during 3 min with HMEBN (200 µl) under agitation (approximately 80 rpm). The detection of C3b was performed with 50 µL of rabbit anti-human C3b antibody (Sigma: C-6025) 12.6 µg/µl, diluted at 1∶1000 in HNB. The incubation with the antibody was performed during 30 min at room temperature under agitation. The non-adhered antibodies were washed with HMEBN as described above. For C3b detection, each well was incubated with 50 µL of monoclonal anti-rabbit antibody conjugated with peroxidase (Sigma: A2074) diluted at 1∶1500 in HNB during 30 min under agitation. After washing, the wells were rapidly filled with 200 µL of the substrate OPD-H_2_O_2_ (1 mg/mL O-Phenylene-Diamine (1,2-Benzenediamine) [Sigma: P-9029]) dissolved in 50 mM sodium citrate/HCl buffer, pH 5.5 containing 0.075% H_2_O_2_). Readings were performed during 5 min at 450 nm in a microplate reader (Bio-Rad, Benchmark with the software Microplate Manager 5.2, 2002) every 30 sec (kinetic mode) at room temperature. The maximal velocity (i.e. the rate of absorvance increase) was calculated from the raw data, by the software Microplate Manager 5.2, 2002, and the generated data used for statistic calculations.

#### Classical pathway assay [modified from ref. [16]]

A 96 well microplate (COSTAR® code 9017) was sensitized during 12 hr at room temperature, inside a humid chamber, with 2 µg of purified human IgG, in 50 µL of sodium carbonate/bicarbonate buffer (15 mM Na_2_CO_3_, 35 mM NaHCO_3_, pH 9.6). Any protocol for the purification of human IgG using Protein A Sepharose affinity could be used to obtain total IgG [44]; however, it is necessary to add 0.88 M NaCl in the sample applied on the Protein A Sepharose column to avoid co-purification of C1q [16], [45]. After purification, the antibody solution was concentrated by ultrafiltration (VIVASPIN 20 ultrafilter, Aldrich code Z614599-48EA) and aliquots were stoked at −80°C after spectrophotometric dosage of protein [46].

The sensitized wells were blocked with 200 µL of LTN solution (0.1% non-fat dry milk in 10 mM TRIS-base/HCl buffer containing 140 mM NaCl, pH 7.4) during 30 min under agitation at room temperature. Wells were blocked for a second time in the same conditions with LTNTC solution (0.1% non-fat dry milk in 10 mM TRIS-base/HCl buffer containing 140 mM NaCl, 5 mM CaCl_2_ and 0.05% Tween-20, pH 7.4).

One microliter of normal human sera was pre-mixed inside microcentrifuge tubes, immediately before the assays, to adequate amounts of the samples containing the inhibitors (salivary gland extracts, intestinal contents, haemolymph or intestinal microvillosities/perimicrovillar membranes preparation). The final volume was adjusted to 100 µL with HNCM. Controls were prepared in separate tubes, by adding sera and HNCM solution. Each one of these treatments was carried out in triplicate. Each 100 µL preparation was then transferred to wells sensitized with human IgG and incubated 30 min at 37°C under agitation. After incubations, each well was washed with 200 µL HNCM as described above.

C4b and C3b detection was performed with rabbit anti-human C4b (5.9 µg/µL) (SIGMA: C3402) or rabbit anti-human C3b (12.6 µg/µL) antibodies, respectively. The detection of C1q component was performed with goat anti-human C1q antibodies (33 µg/µL) (SIGMA: C3900). The anti-C4b and anti-C3b antibodies were diluted at 1∶1000 in NH-1 solution (10 mM HEPES/NaOH, 140 mM NaCl, pH 7.4). The anti-C1q antibody was diluted at 1∶1000 in NH-2 solution (10 mM HEPES/NaOH, 860 mM NaCl, pH 7.4). Fifty microliters of each diluted antibody was transferred to the respective wells and incubated for 30 min at room temperature under agitation. The non-adhered antibodies were washed with 200 µL of HNCM. For C4b or C3b detection, each well was incubated with 50 µL of monoclonal anti-rabbit antibody conjugated with peroxidase (Sigma: A 2074) diluted at 1∶1500 in HN-1 during 30 min, at room temperature, under agitation. For C1q detection, the same procedure was performed but, using 50 µL of rabbit anti-goat antibody conjugated with peroxidase (Calbiochem: 401504). Washing, color development and readings were performed as described for the alternative pathway assay.

### Statistic calculations and presentation of experimental data

In at least three independent experiments, the averages of the maximal velocities (the rate of absorvance increase) obtained for each concentration of inhibitor (assayed in triplicate) were compared to their respective controls using the paired T test of Student. The calculations were performed using the software GraphPad 3.0. Only results with p<0.05 were considered different from controls. In order to be presented, the data from the experiments were transformed in percentage of inhibition in relation to the respective controls. The results here presented refer to the averages from these percentages±standard deviation. In the tables, asterisks indicate treatments considered statistically different in relation to their controls.

### Determination of the *T. brasiliensis* anterior midgut pH and study of the pH influence in the operation of the human complement system

The *T. brasiliensis* anterior midgut pH was determined by using liquid membrane microelectrodes according to the methodology described by Santos et al. [14]. The influence of the pH in the performance of the classical and alternative pathways was investigated in the pHs 6.0 to 8.5 with 0.5 pH unit intervals. For such, the techniques described here for the classical and alternative pathways were slightly modified. The serum was combined in microcentrifuge tubes with HMEBN (alternative pathway) or HNCM (classic pathway) with pH values adjusted from pH 6.0 to 8.5. Complement inhibitors were not added in these experiments and the performance of the complement system was inferred by measuring C3b deposition as described. The readings in different pHs were compared with the ones obtained in pH 7.4. Statistical calculations were performed as described.
